# Debromoaplysiatoxin as the Causative Agent of Dermatitis in a Dog after Exposure to Freshwater in California

**DOI:** 10.3389/fvets.2017.00050

**Published:** 2017-04-06

**Authors:** Birgit Puschner, Adrienne C. Bautista, Chris Wong

**Affiliations:** ^1^Department of Molecular Biosciences, School of Veterinary Medicine, University of California, Davis, CA, USA; ^2^California Animal Health and Food Safety Laboratory System, School of Veterinary Medicine, University of California, Davis, CA, USA; ^3^VCA Sacramento Veterinary Referral Center, Sacramento, CA, USA

**Keywords:** dog, cyanobacteria, cyanotoxins, debromoaplysiatoxin, *Lyngbya* sp., skin irritation, toxicosis

## Abstract

Contamination of recreational waters with cyanobacterial toxins continues to increase and presents a risk to animals and humans. Although cases of acute hepato- and neurotoxicoses in dogs following cyanotoxin exposure exist, no reports of skin-related reactions in dogs exist. A 5-year-old female spayed 34 kg Bracco Italiano was initially presented for rapid onset of severe pruritus and urticaria. Marked excoriation and erythema were noted over the chest and neck, while urticaria was noted in the inguinal regions and ventral abdomen. Initial basic dermatology work-up excluded parasitic, fungal, and bacterial organisms. Due to the severity and progression of urticaria, the dog received IV dexamethasone and IM diphenhydramine. Improvement of the urticaria and the dog’s clinical status was noted over the next 45 min. Assessment of the dog’s environment revealed access to a lake on the property with visible algal bloom. Water from the lake was submitted for toxicology testing and revealed the presence of debromoaplysiatoxin. Access to the lake was discontinued and follow-up evaluation over the next few weeks revealed a complete resolution of the skin irritation. To the authors’ knowledge, this is the first case report of debromoaplysiatoxin exposure in a dog after swimming in cyanobacteria-contaminated water. Veterinarians should recognize the potential harm that contaminated waters may cause in terms of dermal, hepatic, and neurological conditions. In addition, more prudent oversight of contaminated recreational waters is recommended for animals and humans to prevent adverse events and intoxications.

## Introduction

Cyanobacteria poisoning in dogs is not a new phenomenon. Most reports document exposure to hepatotoxic microcystins (1–4) and neurotoxic anatoxin-a (5, 6). In contrast, documentation of the irritant and allergenic effects of cyanotoxins is limited with most reports referring to studies in laboratory animals (7, 8) or epidemiological studies in humans (9–12). Although skin irritation after exposure to recreational waters is routinely mentioned as a health risk, a majority of effort has been focused on the association between total coliform, fecal coliform, and *E. coli* and skin-related symptoms (13) and not cyanobacteria. Dermatotoxins produced by cyanobacteria have been linked to outbreaks of skin irritation and include aplysiatoxins, debromoaplysiatoxins, and lyngbyatoxins. However, cyanobacterial dermatotoxins were not widely recognized until the late 1950s when an epidemic of acute contact dermatitis was reported in bathers along the beaches of Oahu, Hawaii (14). Exposure to *Lyngbya majuscula* was suspected and studies using purified extracts from *L. majuscula* positively identified exposure to this cyanobacterium as the inciting cause (15). Nevertheless, it would take more than a decade before the exact toxin, debromoaplysiatoxin, was isolated and identified (16). Unfortunately our understanding of cyanobacteria-associated dermatologic illnesses from exposure to freshwater is lacking with most acute dermatitis studies focusing on marine cyanobacteria such as the Hawaii epidemic.

A 2006 review of anecdotal and case reports and epidemiological studies of recreational and occupational exposure to freshwater cyanobacteria found that the true incidence of acute cyanobacteria-associated illness is unknown, likely due to under-recognition and under-diagnosis by healthcare providers (10). As our understanding of the toxins associated with cyanobacteria increase and our ability to detect the various toxins in biologic samples expand, linking exposure to illness will become easier. In addition, given the association between increasing temperatures and increased frequency of cyanobacteria blooms around the world, the incidence of cyanobacteria-associated illness will likely increase (17). Changing climates and anthropogenic activities are also likely to have an impact; therefore, healthcare providers and veterinarians will have to be vigilant when it comes to evaluating all aspects of adverse outcomes to cyanotoxins from recreational waters.

## Case Presentation

Within 24 h of swimming in a lake in Northern California, a 5-year-old, 34-kg, spayed female Bracco Italiano developed severe pruritus, urticaria, and malaise and presented to the VCA Sacramento Veterinary Referral Center (SVRC) for evaluation. On evaluation, a large area of excoriation with erythema was noted over the cranial chest and at the base of the neck. In addition, the pinnae were erythematous and there was frequent head shaking by the dog. On otoscopic examination, the tympanic membranes were intact with only a small amount of cerumin noted; no erythema was seen within the canals. The dog’s vulva was also thickened and erythematous with urticaria noted on the skin adjacent. Urticaria was evident in the inguinal regions and ventral abdomen and appeared to progress during the physical evaluation. A skin scraping of the cranial thorax revealed no evidence of parasites. An impression smear, also of the cranial thorax, revealed degenerate neutrophils, a few eosinophils, and no bacteria or fungal organisms. No fungal organisms or bacteria were found on cytology of the ear.

Due to the progression of urticaria over the inguinal region, ventral abdomen, and shoulders during the initial evaluation, a hypersensitivity reaction to an unknown stimulus was suspected. The dog was given dexamethasone SP (0.02 mg/kg IV) and diphenhydramine (2 mg/kg IM) and kept for observation for the next 45 min. During that time, the urticaria appeared to be resolving, although there was still some residual erythema along the cranial thorax and the head shaking continued. Concern by the owner for the possibility of blue-green algae intoxication prompted the collection of blood for selected chemistries, CBC, and venous blood gas analysis. Abnormalities were only identified on the CBC and consisted of a mild neutrophilia (12,940 cells/μL; ref range: 3,000–10,500 cells/μL). The dog was discharged on cephalexin (1000 mg PO q 12 h × 14 days), prednisone (30 mg PO q 12 h for 7 days, followed by tapering to q 48 h for 7 days), and diphenhydramine (75 mg PO q 8–12 h PRN × 5 days). Referral to a dermatology clinic was also recommended if the erythema and head shaking continued. Overnight, the dog vomited several times and developed diarrhea. She was presented to VCA SVRC the next morning and continued to vomit bile and was inappetent. On physical examination, the dog was alert, eupneic, dehydrated, and the abdomen palpated as non-painful. Due to the recent history of hypersensitivity reaction, GI shock was considered the most likely cause for vomiting and diarrhea; however, other underlying causes were also considered especially because of the dog’s previous history of eosinophilic and lymphocytic-plasmacytic enteritis. The owner elected inpatient care consisting of the administration of LRS (on a 5% rehydration rate over 6 h, IV), famotidine (1 mg/kg, IV, q 24 h), metronidazole (10 mg/kg, IV, q 12 h), dolasetron (0.6 mg/kg, IV, q 24 h), and diphenhydramine (2 mg/kg, IM, TID). She was also placed on NPO before being referred to the VCA critical care unit for further monitoring, where clinical improvement was noted over the next 48 h. The dog was sent home after 2 days of hospitalization. Follow-up evaluation after 1 week revealed a complete resolution of clinical signs.

**Figure 1:**
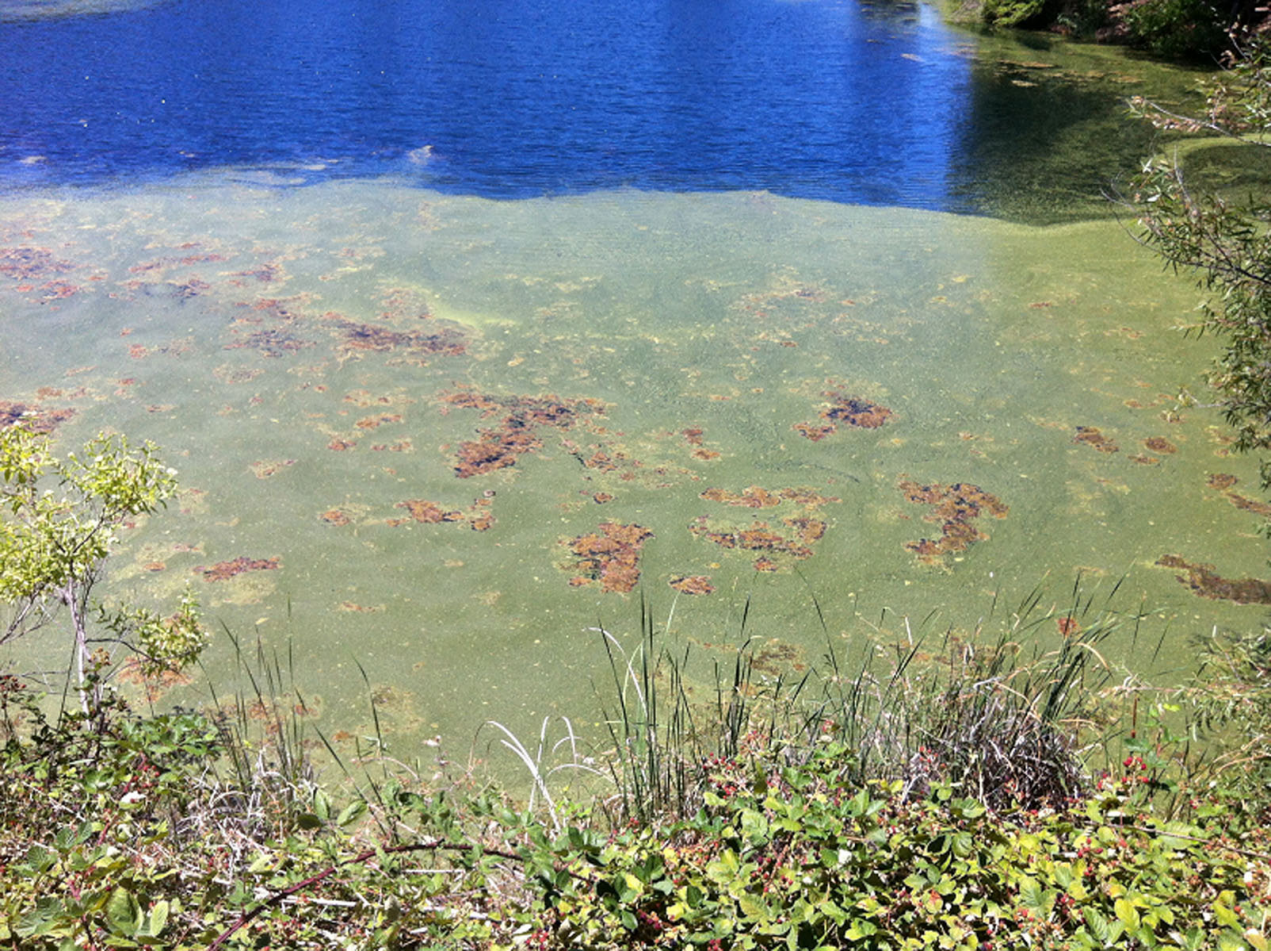
**Mat-forming algal bloom along the shore of the lake the dog accessed**.

**Figure 2:**
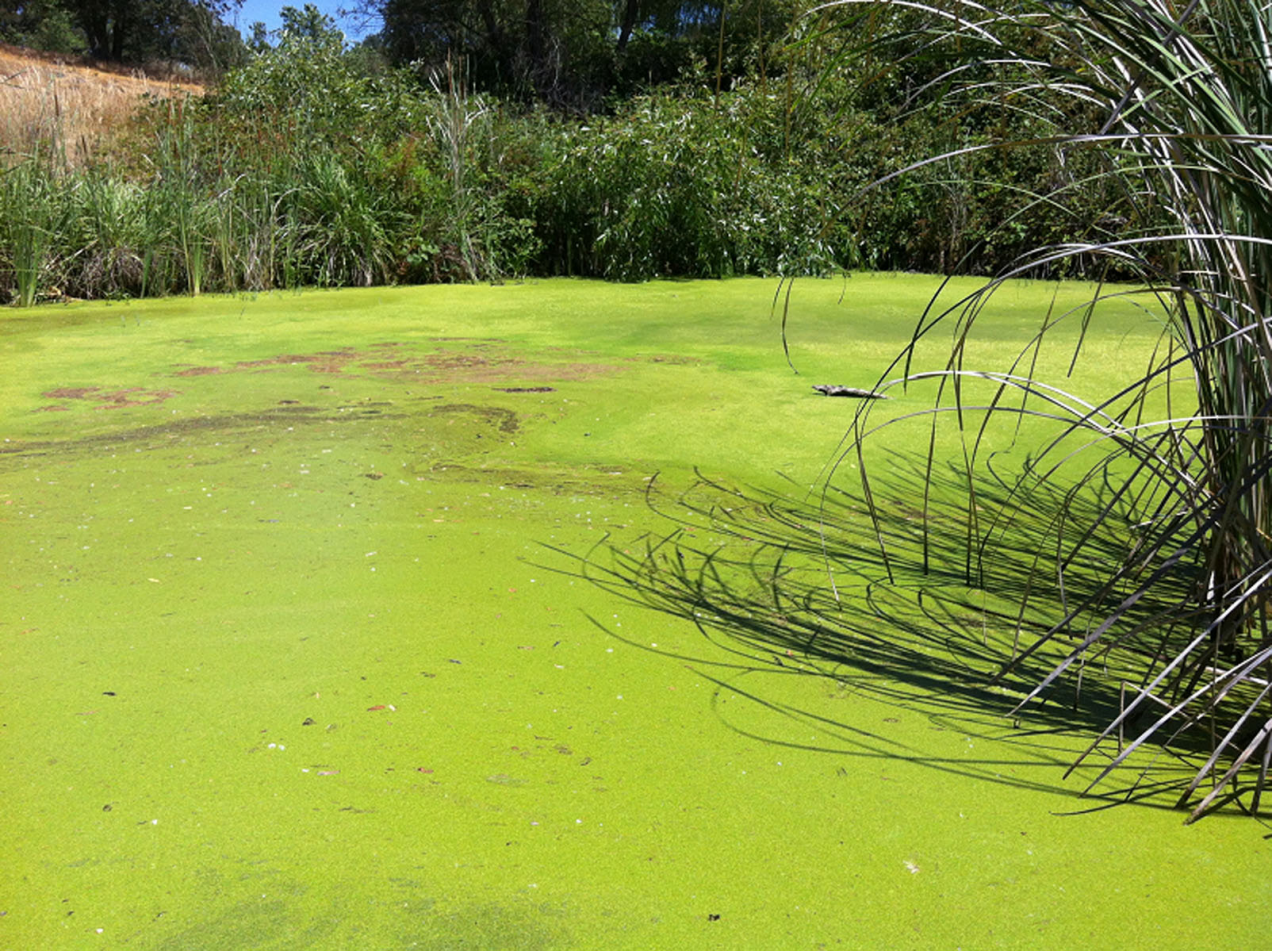
**Lake surface covered with a dense growth of *Wolffia columbiana***. *W. columbiana* is a small, free-floating aquatic plant commonly known as watermeal and native to California. Watermeal is non-toxic and thrives in nutrient-rich water, conditions suitable for cyanbacteria growth and toxin production.

**Figure 3:**
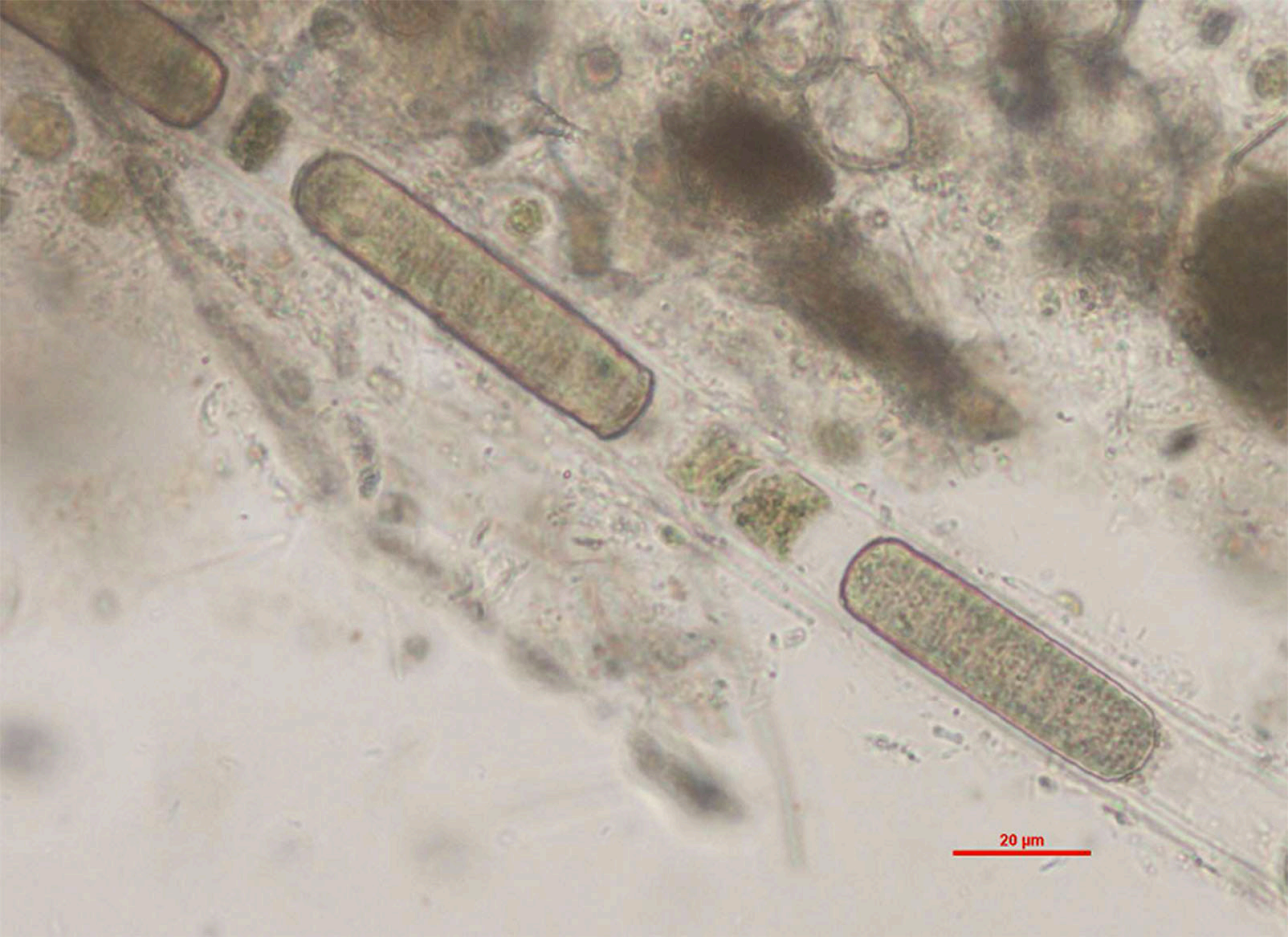
***Lyngbya* sp. identified in the lake water**. Filamentous cyanobacteria with a thin, colorless sheath. Wet mount of lake water, ×400 (scale bar = 20 μm).

**Figure 4:**
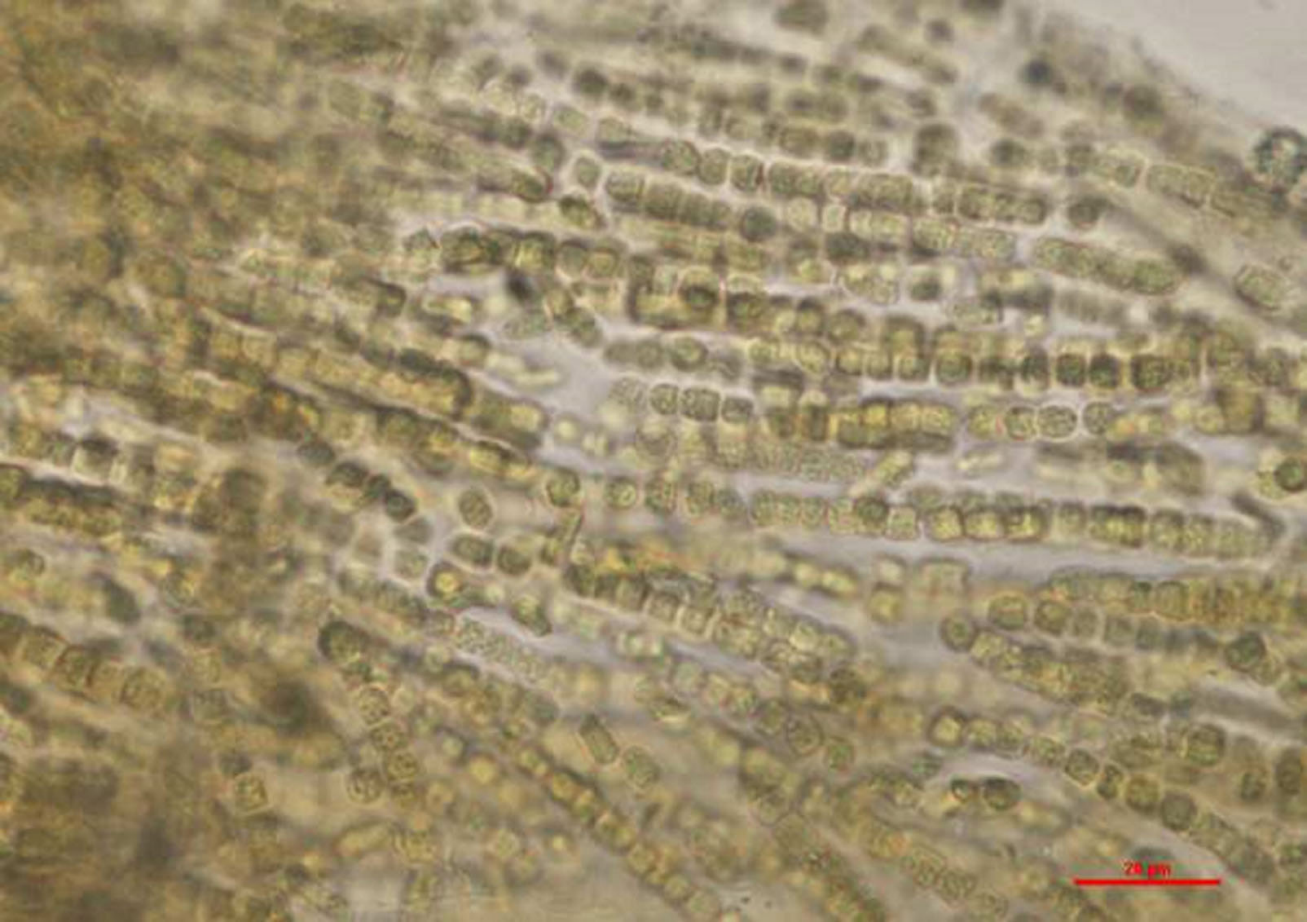
***Anabaena*/*Trichormus* sp. identified in the lake water**. Only one colony of filaments was observed in the sample. Akinetes, important characteristics morphological features were not present which prevented further identification to the lowest possible taxonomic level. Wet mount of lake water, ×400 (scale bar = 20 μm).

Careful evaluation of the dog’s environment revealed access to several lakes with visible algal bloom (Figures 1 and 2). Water from the lake the dog had access to prior to developing illness was submitted for phycological and toxicological evaluation. Microscopic observation of the water sample (Green Water Laboratories/Cyanolab, Palatka, FL, USA) showed a dominant presence of a tiny vascular plant *Wolffia columbiana* (common name: watermeal), a high density of motile bacterial rods and bacterial filaments, and an abundant filamentous cyanophyte called *Microchaete cf. uberrima*. The water sample also contained green algae (*Chlorophyta*) and diatoms (*Bacillariophyta*). Specific to cyanobacteria, the water sample contained *Microchaete cf. uberrima, Lyngbya* sp. (Figure 3), *Pseudanabaena* sp., *Anabaena*/*Trichormus* sp. (Figure 4), *Microchaete* sp., *Scytonema* sp., *Calothrix* sp., and *Aphanothece elabens*. Amongst the cyanobacteria identified, only *Anabaena*/*Trichormus* sp. and *Lyngbya* sp. were of toxicological significance. Unfortunately, positive species identification for *Anabaena*/*Trichormus* sp. and *Lyngbya* sp. could not be achieved from collected specimens.

The water samples were also analyzed for cyanotoxins by LC-MS/MS (Green Water Laboratories/Cyanolab, Palatka, FL, USA and the California Animal Health and Food Safety Laboratory, University of California, Davis, CA, USA). The water contained 3.8 μg/g debromoaplysiatoxin [limit of detection (LOD) 0.3 μg/g]. The water also contained a low concentration of anatoxin-a that was estimated to be 1 μg/L. The water contained no aplysiatoxin (LOD 0.5 μg/g), lyngbyatoxin-a (LOD 0.2 μg/g), or cylindrospermopsin (LOD 1 μg/L).

## Discussion

The current case study confirms a case of debromoaplysiatoxin poisoning in a dog following access to contaminated lake water and highlights the potential for serious skin irritation after cyanotoxin exposure. While most of the toxic cyanobacteria are freshwater species, the first documentation of algal toxin-induced dermatitis dates back to 1958 when swimmers along the beaches of Oahu, Hawaii, developed acute, vesicular contact dermatitis (14). A similar outbreak in 1976 again on the shores of Oahu, Hawaii was referred to as “swimmers’ itch” (18). In the past decade, numerous outbreaks in swimmers have occurred in Australia and Hawaii (19, 20). Clinical findings in people include gradual itching and burning progressing to reddening and swelling of the skin with blister formation especially in areas covered by a bathing suit, where the seaweed/toxins are trapped. Desquamation of blisters can lead to skin erosions. The dermatitis typically resolves completely within 1 week. In more severe cases, symptomatic treatment with analgesics, antihistamine, and steroids is provided. *Lyngbya* dermatitis can develop after only minutes of exposure to contaminated waters; airborne contact dermatitis on faces has also been noted during weather patterns with strong winds. The dog in this case developed severe pruritus and urticaria within 24 h of swimming in contaminated lake water. Prompt treatment with dexamethasone and diphenhydramine prevented progression to a more severe dermatitis. Within 1 week, the dog’s skin condition had resolved completely.

Isolation of the actual dermatotoxins from *L. majuscula* and from the digestive gland of the sea hare *Stylocheilus longicauda* occurred in the early 1970s (21, 22). Subsequent toxicity studies with debromoaplysiatoxin in laboratory animals and human volunteers confirmed this compound to be a potent primary skin irritant (23). Mice and rabbits developed erythema, edema, and sloughing of skin within 6 h of topical application of a 0.05% debromoaplysiatoxin solution. Human volunteers described tingling and burning sensation after application and developed an irritant pustular folliculitis between 6 and 12 h of topical administration that required up to 10 days to resolve. Debromoaplysiatoxin was subsequently isolated from other tropical marine cyanobacteria including *Lyngbya gracilis, Oscillatoria nigroviridis*, and *Schizothrix calcicola* (16). In the freshwater environment, the filamentous, benthic cyanobacterium *Lyngbya wollei* is now the cause for common proliferations in lakes and rivers from the St. Lawrence/Great Lakes basin to Florida (24). In the lake sample collected for this case work-up, the presence of *Lyngbya* sp. was confirmed by phycological evaluation. Unfortunately, only one colony of filaments was observed in the presence, which did not allow for species identification. The finding of *Lyngbya* sp. in the lake water is not surprising, considering *L. wollei* has been recorded in three sites of California streams (25). *L. wollei* has the ability to produce a wide range of toxins including paralytic shellfish toxins, and hepatotoxic cylindrospermopsin and its derivatives (26). Currently, it is unclear whether *L. wollei* is capable of producing dermatotoxins. Interestingly, most recent review articles provide in-depth information on the neuro- and hepatotoxicity of cyanotoxins but highlight the need for further examination of dermatotoxins because they bear major potential public health consequences for recreational users, while there are very limited toxicity and geographic distribution data available (27–29). In addition to debromoaplysiatoxin, aplysiatoxin and lyngbyatoxins have been associated with skin irritation (30). A debromoaplysiatoxin-producing *Lyngbya* bloom that occurred in the Homosassa River in 2008 was hypothesized as the cause for ulcerative dermatitis in manatees (31). Thus, while debromoaplysiatoxin is predominantly detected in marine water, one report documents its occurrence in freshwater ecosystems in Florida. Our report adds additional occurrence data of debromoaplysiatoxin in freshwater and is the first documentation of debromoaplysiatoxin in freshwater from a Northern California lake resulting in skin irritation in a dog. Debromoaplysiatoxin produced dermatitis on the murine ear at 0.005 nmol (equivalent to 2.7 ng) per ear (32). Considering an estimated biomass of 30 mg/L dry weight in the lake water, 1 L of water would have contained 114 ng of debromoaplysiatoxin. Based on the limited toxicity data available for topical exposure, the concentrations found are considered to be capable of resulting in dermal irritation. The lake water contained no detectable amounts of aplysiatoxin or lyngbyatoxin-a. Debromoaplysiatoxin, aplysiatoxin, and lyngbyatoxins exert their toxic effects by binding and activating protein kinase C isozymes (33). PKC belongs to a family of serine/threonine kinases and plays important roles in cellular signaling transduction for proliferation, differentiation, and apoptosis (34); activation of PKC can induce cutaneous inflammation (35). Debromoaplysiatoxin and its brominated analog aplysiatoxin can also cause diarrhea in animals and humans (36, 37). The dog developed diarrhea and vomiting approximately 48 h after swimming in the lake. These gastrointestinal signs could have been a direct toxic insult of debromoaplysiatoxin on the intestine similar to what has been confirmed after oral administration of aplysiatoxin to mice (38). However, because of the dog’s previous history of eosinophilic and lymphocytic-plasmacytic enteritis, other underlying causes were also considered.

It is important to consider the role of anatoxin-a in the clinical presentation of this dog since anatoxin-a was detected in the lake water sample. Anatoxin-a is a potent cholinergic agonist at nicotinic acetylcholine receptors in neurons and at the neuromuscular junctions and can lead to muscle tremors and, in severe cases, seizures and respiratory paralysis (6, 39). The toxin is produced by many different cyanobacteria including as *Anabaena, Plantkothrix, Oscillatoria, Microcystis, Aphanizomenon, Cylindrospermum*, and *Phormidium* sp. Anatoxin-a has been found in rivers and lakes in CA (39). *Anabaena/Trichormus* sp. was identified in the water sample; due to lack of akinetes in the sample, further identification to the species level was not possible. However, the fact that anatoxin-a was identified in the water sample is a clear indication of the presence of a toxin producer. Fortunately, the dog did not develop neurologic signs, which is likely attributable to exposure to a dose below the toxic threshold. In other reported anatoxin-a poisoning cases, values of anatoxin-a ranging from 0.01 mg/L to 1 mg/L were detected (6, 39). In contrast, an estimated concentration of 1 μg/L was found in this case. This finding highlights the importance of toxicological evaluation of suspect water sample. While phycological examination of a water sample aids in the diagnostic approach of a suspect cyanotoxins exposure, it is important to note that the production of toxins by cyanobacteria is strain specific, and morphological observations alone cannot predict the hazard level. Thus, detection of toxins is needed to confirm a suspect intoxication.

## Concluding Remarks

This study adds an additional toxin, debromoaplysiatoxin, to the list of freshwater toxins resulting in acute illness in animals. The presence of debromoaplysiatoxin in recreational waters has health implications for pets and humans using environments containing high concentrations of this cyanotoxin. Our investigation offers insight into the dermatotoxic risk from cyanotoxin exposure in companion animals, as well as the risk for co-exposure to multiple toxins and a complex clinical presentation. To the authors’ knowledge, this is the first documentation of debromoaplysiatoxin-induced skin irritation in a dog. Owners and veterinarians should recognize the potential harm in allowing dogs to access cyanobacteria-contaminated waters. If adverse effects are noted, access to water must be denied, and supportive care should be initiated. In addition, more prudent oversight of recreational waters is recommended to prevent adverse events/intoxications not only for companion animals but also for humans.

## Author Contributions

AB was responsible for the toxicology case work-up and interpretation of test results. CW performed the clinical work-up and care of the dog. BP drafted the manuscript, mentored AB with the toxicological work-up, and acquired photographic materials. All authors read and approved the final manuscript.

## Conflict of Interest Statement

The authors declare that they have no competing interests. All data generated or analyzed during this study are included in this published article.

## Abbreviations

IM, intramuscular; IV, intravenous; NPO, nil *per os*, withhold oral intake of food and fluids; PO, orally; PRN, pre re nata, when necessary; Q, every.
